# Three-year follow-up of changes of cortical bone thickness after implantation of Endo-Exo-Prosthesis (EEP) for transfemoral amputees

**DOI:** 10.1186/s13018-020-01675-w

**Published:** 2020-05-04

**Authors:** Marcus Örgel, Emmanouil Liodakis, Pratya Jaratjitwilai, Afif Harb, Nils Wirries, Mohamed Omar, Christian Krettek, Horst-Heinrich Aschoff

**Affiliations:** 1 Trauma Department, Hannover Medical School (MHH), Carl–Neuberg–Straße, 130625 Hannover, Germany; 2 Bangkok Hospital Pattaya, Banglamung, Chonburi, Thailand; 3 Orthopaedic Department, DiakovereAnnastift, Hannover, Germany

**Keywords:** Transfemoral amputee, Endo-Exo-Prosthesis, Osseointegration, Transcutaneous osseointegrated prosthetic system (TOPS)

## Abstract

**Introduction:**

Transcutaneous Osseointegrated Prosthetic Systems (TOPS) offer a good alternative for patients who cannot be satisfactorily rehabilitated by conventional suspension sockets. The Endo-Exo-Prothesis (EEP, ESKA Orthopaedic Handels GmbH®, Deutschland) is the most implanted TOPS in Germany. Previous studies have shown that cortical thickness increases after implantation of TOPS. The aim of this study is to determine changes of cortical thickness in relation to the time after implantation of the Endo-Fix-Stem.

**Patients and methods:**

All transfemoral amputees treated by EEP from 2007 to 2013 were operated by the last author of this study. X-ray images of 4 follow-up intervals (postoperative, 3 months, 12 months, 3 years) were analyzed retrospectively. The femoral residuum was divided into 3 sections (proximal, middle, distal) with 2 measuring points in each section: medial and lateral. Cortical thickness was measured at these 6 points and compared at regular intervals using the Friedman test for non-parametric dependent variables.

**Results:**

Thirty-seven patients with 40 implants were included. The average age was 52.2 years (30–79 years). 83.7% of the patients were male. No statistical significance could be shown for any of the measuring points of the femoral residual (proximal medial, proximal lateral, middle medial, middle lateral, distal medial, distal lateral) among the mean values of the cortical thickness at the different follow-up times (*p* > 0.05 for all measuring points). Cortical remodeling processes (> 1 millimeter (mm)) occurred in all implants despite a missing statistical significance. Hypertrophy could be confirmed for 42.5% and atrophy for 37.5%. Twenty percent of the cases showed a parallel occurrence of both entities. Cortical changes greater than 5 mm were only observed at the distal end of the femur.

**Conclusion:**

Even if our results did not show any significant difference, it can be deduced that the osseointegration process leads to a remodeling of the bone structure, both in terms of increased bone formation and bone resorption. However, it has not yet been conclusively clarified which processes lead to hyper- or atrophy. The force transmission between prosthesis and bone and the facultative bacterial colonization of the stoma are still the main factors which may be responsible for the bone remodeling processes.

## Introduction

The rehabilitation of upper and lower limb amputees by transcutaneous osseointegrated prosthetic system (TOPS) has been used in Sweden since 1990 [1] and offers a good alternative for patients who cannot be satisfactorily rehabilitated by conventional suspension sockets [2–9]. Currently, there are a lot of different TOPS available on the market. The Endo-Exo-Prothesis (EEP, ESKA Orthopaedic Handels GmbH®, Deutschland) is the TOPS which has been used most often in Germany since 1999. Approximately, 150 patients have been treated with EEP in Germany [7].

**Fig. 1:**
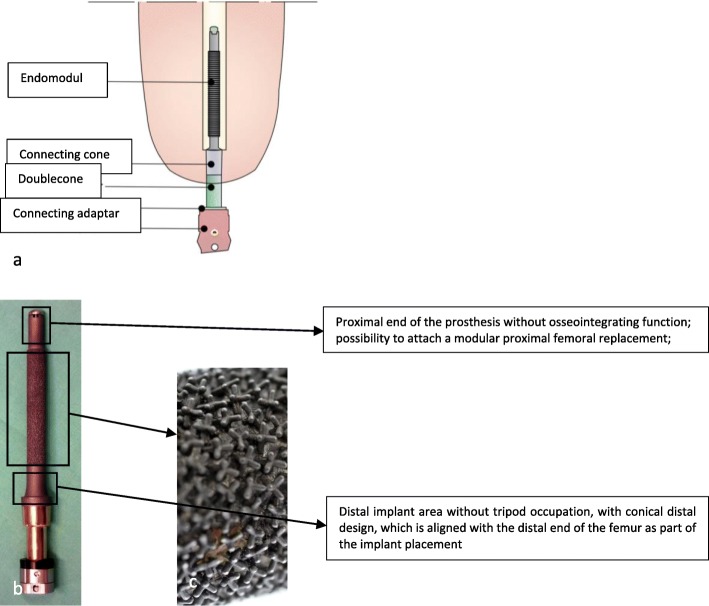
**a** Schematic representation of the Endo-Exo-Prosthetic System. **b** Standard Endo-Fix-Stem with highlighted different areas of the prosthesis. **c** Enlargement of the implant surface of the tripods

Implantation of an EEP is performed as a two-stage procedure in our hospital. At first, the Endo-Fix-Stem is implanted, and then—after an osseointegration period of approximately 6 weeks—a stoma is applied to connect the external module to the inner femoral stem (Fig. 1) [3, 4, 6, 10, 11]. The osseointegration process is of central importance for success of the procedure. Grundei developed a 1st and 2nd generation spongy metal surface for this reason [12, 13]. The second generation represents the surface of the stem used today. This surface consists of tripods with a selectable construction height of 0.65–3 mm. They allow bone ingrowth in a reproducible strong and spatially structured manner. In addition, the three-dimensional surface of the tripods ensures a better distribution of the forces acting on the implant in order to minimize any movements between implant and bone [13]. It is also intended to prevent any formation of soft tissue between implant and bone which has been associated with a higher risk of loosening and intramedullary ascending infections [13–16]. The use of EEP and the interaction between implant and bone lead to an increase in bone areas with higher load absorption, while the cortical structure is reduced in the bone areas which are under less pressure [17–20]. This implant-bone interaction is based on Julius Wolff’s theories [17]. Previous studies have shown that cortical thickness increases after implantation of TOPS [21].

One of the most important aspects for the use of transcutaneous osseointegrated prosthetic systems (TOPS) is the osseointegration of the Endo-Fix-Stem (implant). The entire success of this system depends on the osseointegration. Therefore, the structural changes of the peri-implant bone are essential. The purpose of this retrospective study is to determine changes of cortical thickness in different areas of the femur residuum in relation to the time after implantation of the Endo-Fix Stem.

## Patients and methods

This retrospective cohort study included all transfemoral amputees treated with EEP from 2007 to 2013. Exclusion criteria were death or implant loss. No patient had to be excluded. The amputees were examined for the clinical-radiological follow-up immediately after operation, 3 months, 12 months and 3 years postoperatively. A total of 37 patients (6 females and 31 males) with 40 implanted Endo-Exo-Prosthesis were included. The patients were on average 52.2 (30–79) years old. Digital x-rays in the anterior posterior (a.p.) view were investigated with regard to cortical structural changes of the femur residuum after EEP treatment in four follow-up periods (see above). The x-rays were imported and evaluated with the program—mediCAD (Version 3.50, mediCAD Hectec GmbH Opalstraße 54, 84032 Altdorf near Landshut, Germany). To detect bone changes in the femur, the femur was divided into three sections—a proximal, a middle, and a distal femoral third. After scaling the x-rays, the cortical thickness was determined at the medial and lateral cortical bone, at these segments. The measurements were compared for all follow-up periods. All measurements were performed manually and were carried out once only.

**Fig. 2:**
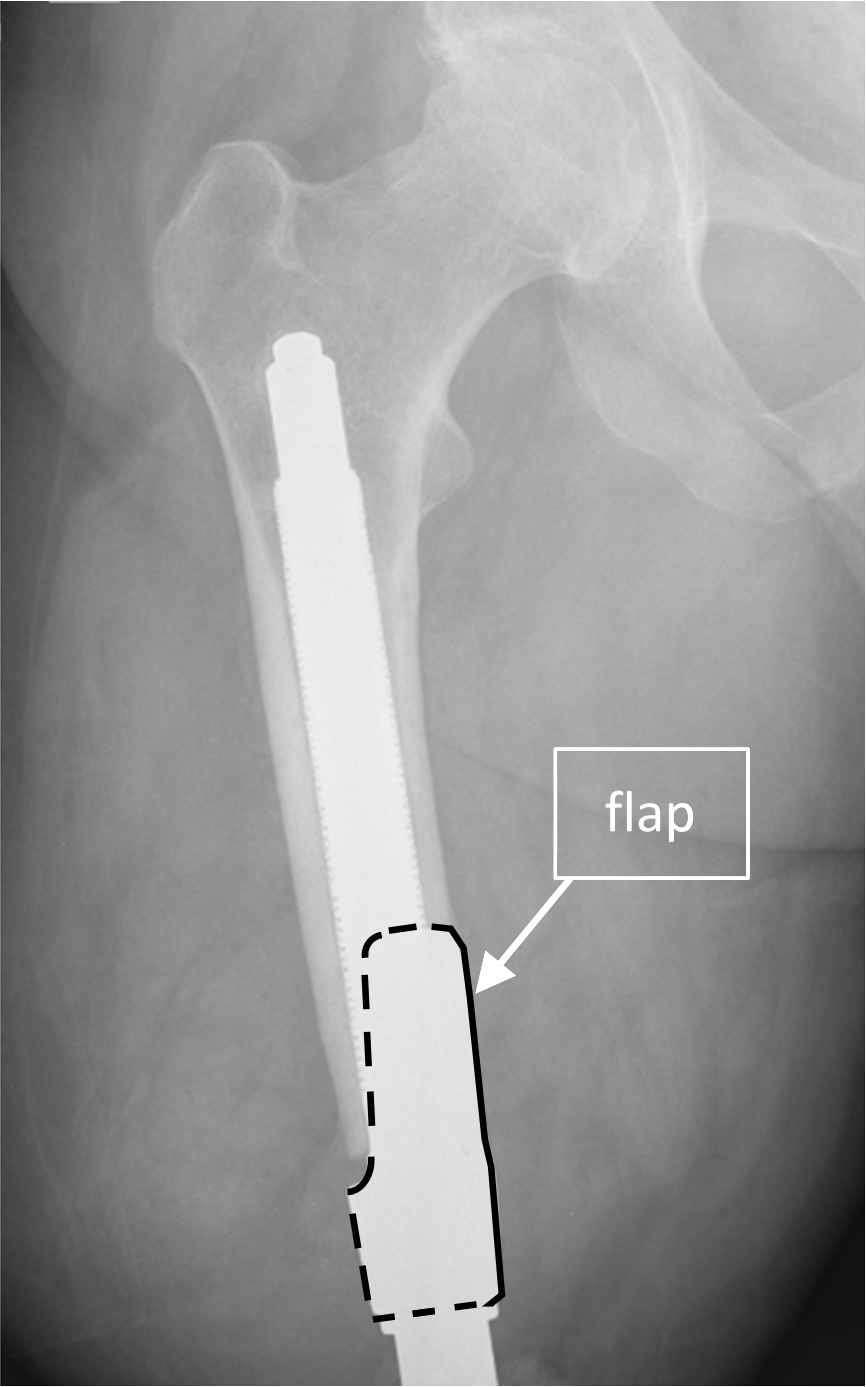
Postoperative x-ray after implantation of an Endo-Fix-Stem. The flap, which was used for the older stems is framed with a dotted and continuous line in black. It was not possible to measure the corticalis thickness in this area

37.8% (14 prostheses) had an implant with a “flap” [4]. This is based on the implant design used until 2009 and partly prevented measurements in the distal femoral third (Fig. 2).

Continuous variables were presented as mean ± standard deviation, while categorical variables were presented as absolute values and percentages (%). Continuous variables were verified for normal distribution using the Shapiro-Wilk test. Differences for dependent, not normally distributed continuous variables (corticalis thickness postoperatively, 3 months, 12 months, 3 years) were compared using the Friedman test, while categorical variables were compared using the Pearson chi-square test.

A two-tailed *p* value of ≤ 0.05 was considered to be statistically significant. The SPSS 23.0 program (SPSS Inc., Chicago, IL) was used for statistical analyses.

## Results

**Fig. 3:**
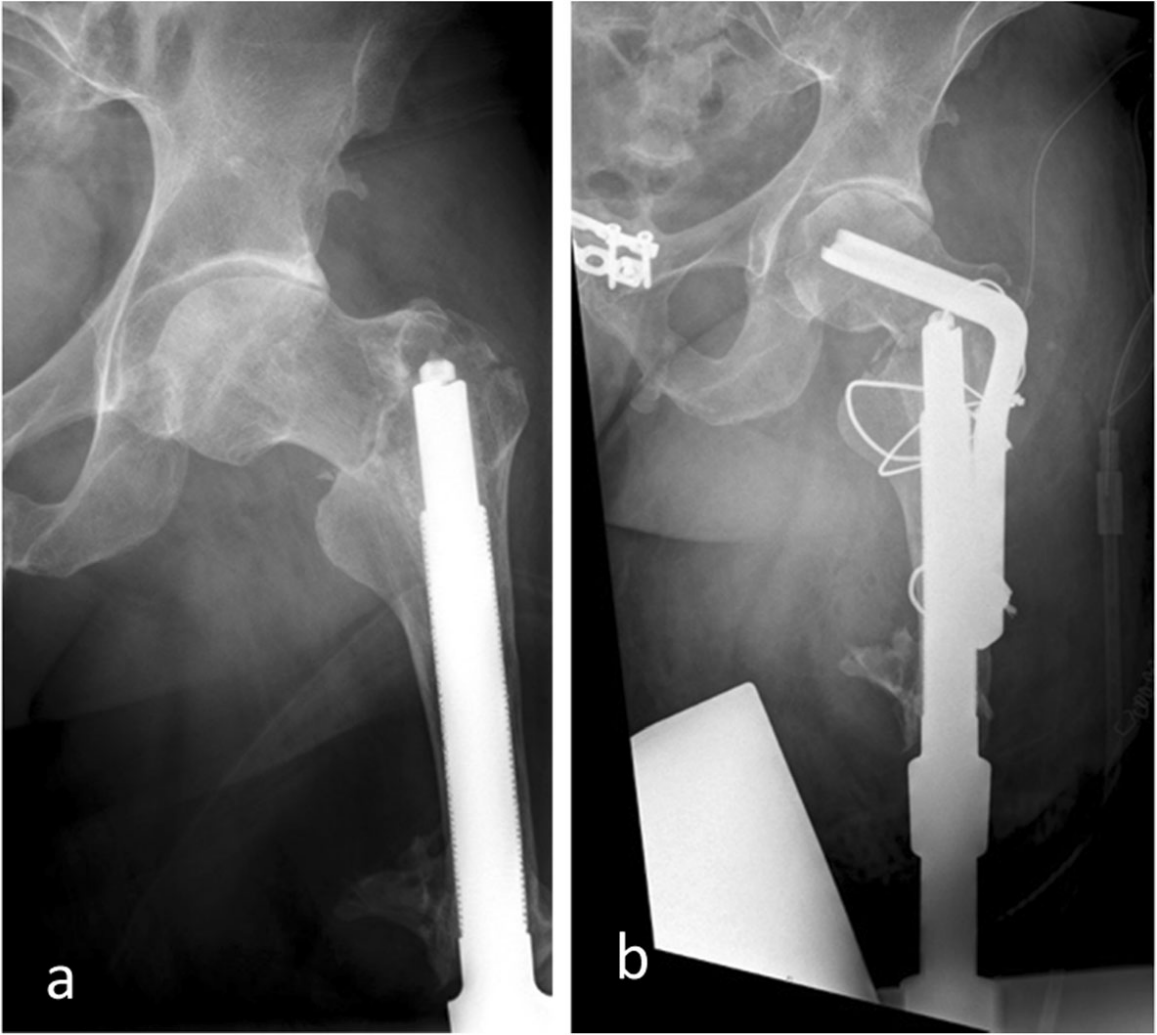
**a** Periprosthetic fracture in left proximal femur after implantation of an Endo-Exo-Prosthesis. **b** Treatment with a 95^o^ angled blade plate

The study included 37 patients with 40 implants. Patient demographics and implant characteristics can be found in Table 1. Three patients suffered from periprosthetic fractures (Fig. 3), which could be treated by open reduction and internal fixation with an angled plate. After 3 months, the patient could be mobilized in a standing and walking position. No further complications occurred during the follow-up periods.

**Table 1:** Patient demographics and implant characteristics

Number of patients (n)	37
Number of implants (*n*)	40
Age (Ø)	52.2
(min.-max.)	(30–79)
Prosthesis diameter in mm (Ø)	17
(min.-max.)	(14–20 mm)
Prosthesis length in mm (Ø)	168
(min.-max.)	(140–180)
Implant side	
Right (*n*)	18
Left (*n*)	22
(Bilateral (*n*))	(3)
Sex	
Male (*n*)	31
Female (*n*)	6
Prosthesis with a flap (*n*)	14

**Fig. 4:**
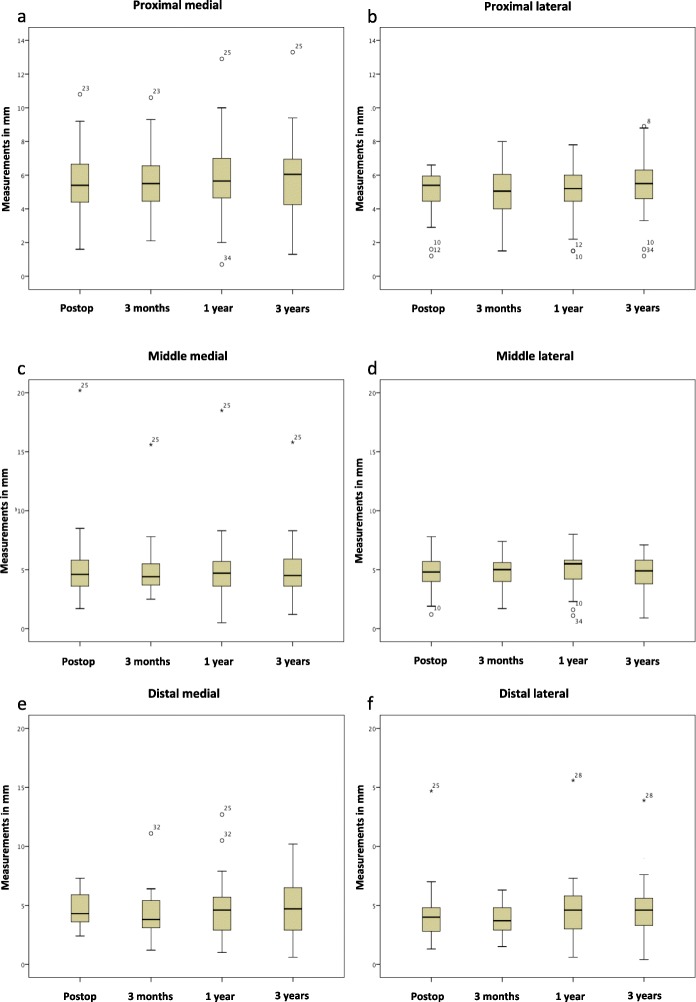
**a**–**f** Graphical representation of the box plots of all measurements at the time of investigation: postoperatively, 3 months, 1 year, and 3 years. o and * are statistical runaways

Statistical mean values including standard deviation and *p* values of the femoral cortical measurements are shown in Table 2. Continuous variables are presented with mean ± standard deviation. In addition, the box plots of the measurements at the follow-up times are shown in Fig. 4.

**Table 2:** Mean femoral corticalis thicknesses (± standard deviation) at the different follow-up periods

Follow-up	Post-op (*n* = 40)	3 Months (*n* = 40)	12 Months (*n* = 37)	3 Years (*n* = 30)	*p* Wert
Mean prox. medial in mm (± standard deviation)	6.92 (± 3.27)	7.16 (± 3.22)	6.54 (± 3.51)	4.59 (± 3.41)	0.692
Mean prox. lateral in mm (± standard deviation)	6.21 (± 2.45)	6.33 (± 2.45)	6.29 (± 3.06)	4.60 (± 3.77)	0.283
Mean middle medial in mm (± standard deviation)	6.31 (± 3.55)	6.45 (± 3.18)	6.21 (± 3.35)	4.28 (± 3.11)	0.745
Mean middle lateral in mm (± standard deviation)	6.42 (± 2.16)	6.52 (± 1.59)	6.47 (± 2.01)	4.42 (± 3.18)	0.105
Mean distal medial in mm (± standard deviation)	6.61 (± 6.83)	6.86 (± 6.48)	6.44 (± 6.39)	3.99 (± 4.61)	0.289
Mean distal lateral in mm (± standard deviation)	5.23 (± 2.75)	5.82 (± 2.33)	7.43 (± 9.53)	4.65 (± 4.23)	0.179

**Fig. 5:**
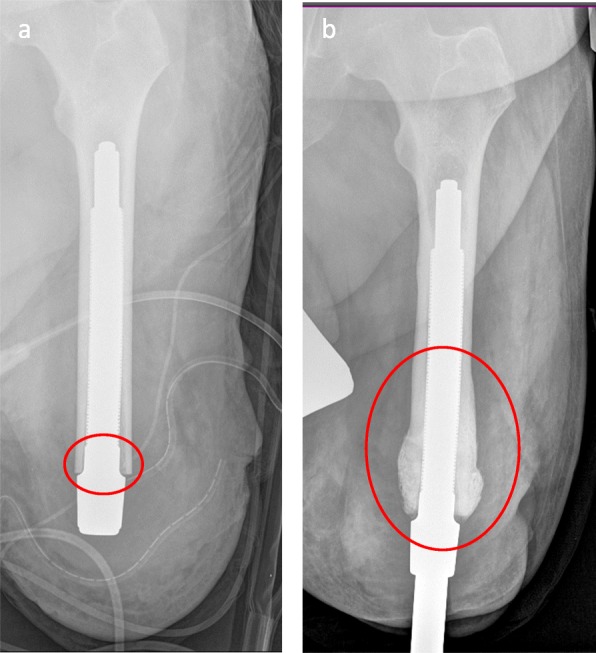
**a**, **b** Postoperative x-ray after Endo-Exo-Prosthesis. The area marked with a red circle (small) depicts the bone-implant transition area that is not covered with tripods. **b** Example of osseous hypertrophy at distal end of femur in the 3-year follow-up including the implant transition area that is not covered with tripods (big red circle)

All 40 implants (100%) showed at least medial and/or lateral osseous structural changes (> 1 mm) in one of the follow-up examinations. Fifteen implants (37.5%) showed distal atrophy of the cortical bone, 17 implants (42.5%) showed osseous hypertrophy (Fig. 5), and eight implants (20%) showed both atrophy/traction and hypertrophy in the distal femoral third region.

A comparison of the cortical thickness between the postoperative and the three-year x-ray control revealed a measurement difference of a hypertrophy of > 5 mm two times distal-medially and three times distal-laterally. In the middle (medial and lateral) and proximal (medial and lateral) measurement point, none of the cases showed a measurement difference of > 5 mm. A measurement difference of 2–5 mm was observed six times distal-medially and seven times distal-laterally. A measurement difference of 2–5 mm occurred five times proximally medially and three times proximally laterally. These measurement results were recorded five times in the mid-medial and three times in the mid-lateral area. The measurement results were not statistically significant. The deviation of the case number at the time of the 3-year control (*n* = 30) from the total number (*n* = 40) was due to the absence of the patients at the follow-up examinations or the use of the abovementioned flap.

## Discussion

The results of our study showed hypertrophy and atrophy changes of the cortical bone in 100% of the investigated cases. These changes were not statistically significant. This partly corresponds to the results of other studies about this topic performed with Osseointegrated Prostheses for Rehabilitation of Amputees (OPRA) and osseointegration prosthesis (OIP) [21–23].

**Fig. 6:**
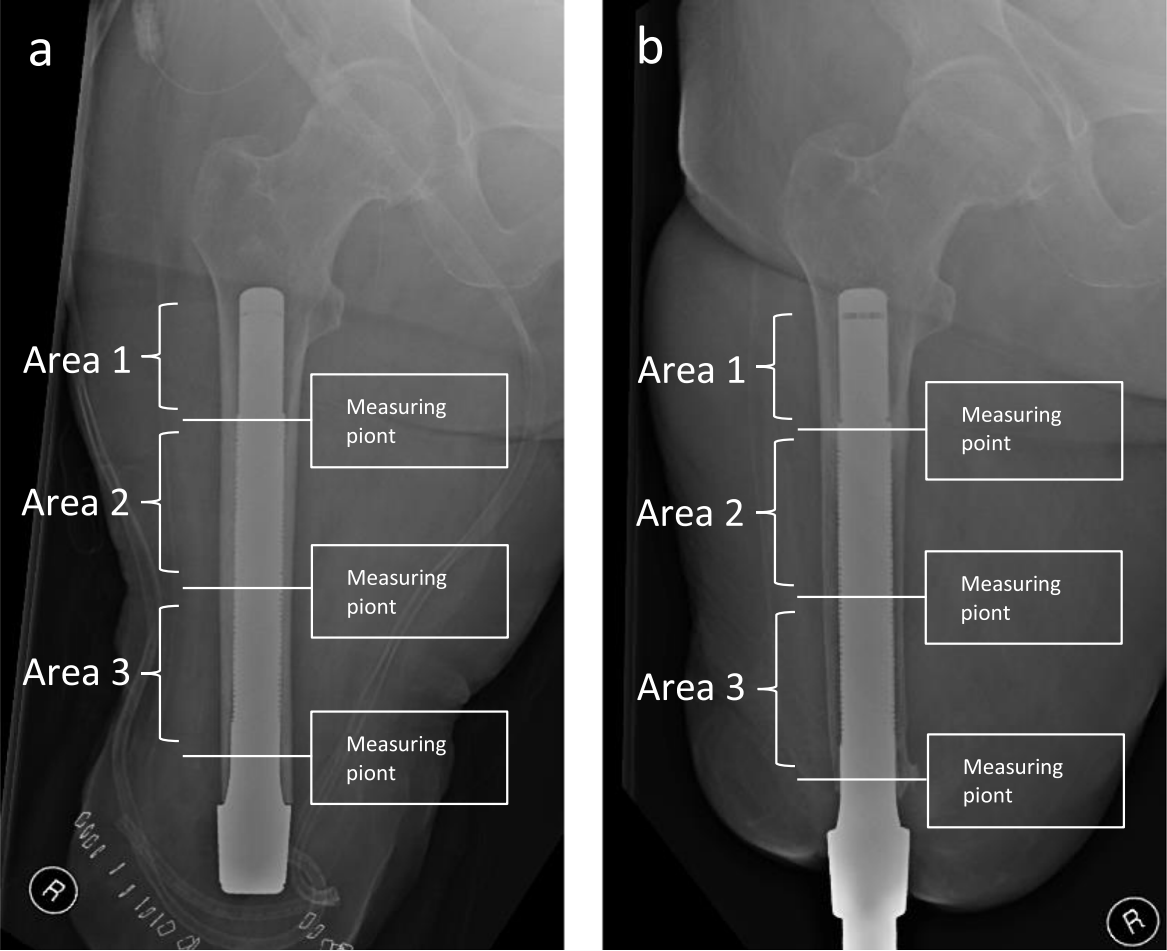
**a**, **b** Postoperative x-ray after implantation of an Endo-Fix-Stem, right thigh, and residual femur; 3-year follow-up after stem implantation, with medial and lateral atrophy of the distal femur. Additionally, **a** and **b** show the division of the femur into three areas

If we compare our results with those from the working group of Haket et al [21], we find an increase in cortical thickness of > 5 mm in five measurements and an increase in cortical thickness between 2–5 mm in 21 measurements in the 3-year control group, but without statistical significance (*n* = 30). However, the working group in the Netherlands used the OIP. The measurement points were the same we used: proximal, in the middle, and distal. They were able to show a statistically significant increase of a maximum of 1.08 mm (*p* = 0.021) (mean value 0.6 mm, *p* = 0.020) in their 1-year check (*n* = 24) compared to the postoperative femoral cortical thickness, and of a maximum of 0.89 mm (*p* = 0.007) (mean value 0.54 mm, *p* < 0.001) in their 2-year check (*n* = 24) compared to the postoperative period [21]. In addition, they performed a bone density measurement of the femoral neck on both the amputated and contralateral side without any evidence of a significant change in bone density in their follow-up studies compared to the values measured prior to use of OIP [21]. The measurement points defined by Haket et al. were close to ours (Fig. 6). Our investigations showed also that the most noticeable bone remodeling processes took place in the distal femur region. This was also demonstrated by Nebergall et al. [23], who performed an examination of the OPRA system with a 6-month (*n* = 47), 1-year (*n* = 42), 2-year (*n* = 40), 5-year (*n* = 15), and 7-year (*n* = 12) follow-up (*n* = 42). In line with our results, they showed that the most noticeable changes of the bone structure were visible at the distal end of the femur [23]. Similar results were presented by Xu et al. and Tomaszewski et al. in their studies, who conducted simulation trials for the OPRA system and for the OIP [22, 24].

Different theories can be considered for the most obvious changes in the distal femoral area, where the prosthesis leaves the bone tube. By adapting the design of the implant, the cylindrical and slightly curved shape was adjusted to the physiological curve of the femur (approximately 6° curve). The intraoperative contour adaptation of the oval, intramedullary bone tube to that of the implant showed that the criteria for long-distance anchoring are to be preferred. Due to the high primary stability (press-fit anchoring) between implant and bone, which occurs during the first operation, the bone grows through the three dimensionally structured prosthesis surface (osseointegration) and creates a strong connection between bone and prosthesis. Nevertheless, an asymmetric implant abutment can be obtained in comparison with the three-point abutment of the press-fit anchoring of cementless hip joint endoprostheses, with a consecutive rise of the osseous structure in the areas where the prosthesis is anchored in the bone, and a reduction of the bone structure in the less heavily loaded areas. This effect is described in the implantation of cementless hip prostheses, along with the bone changes according to the stress-shielding in the Gruen Zones according to Wolff’s law [17–20, 25–28]. If this theory is applied to the EEP, “fitting errors” could occur during the implantation of the Endo-Fix-Stem, and asymmetrical load distributions between bone and implant could occur at certain points on the intramedullary anchoring path of the prosthesis. According to Wolff’s law and the conditions of stress shielding [20, 26, 29, 30], randomly placed, cortical changes along the entire press-fit anchorage length could occur.

**Fig. 7:**
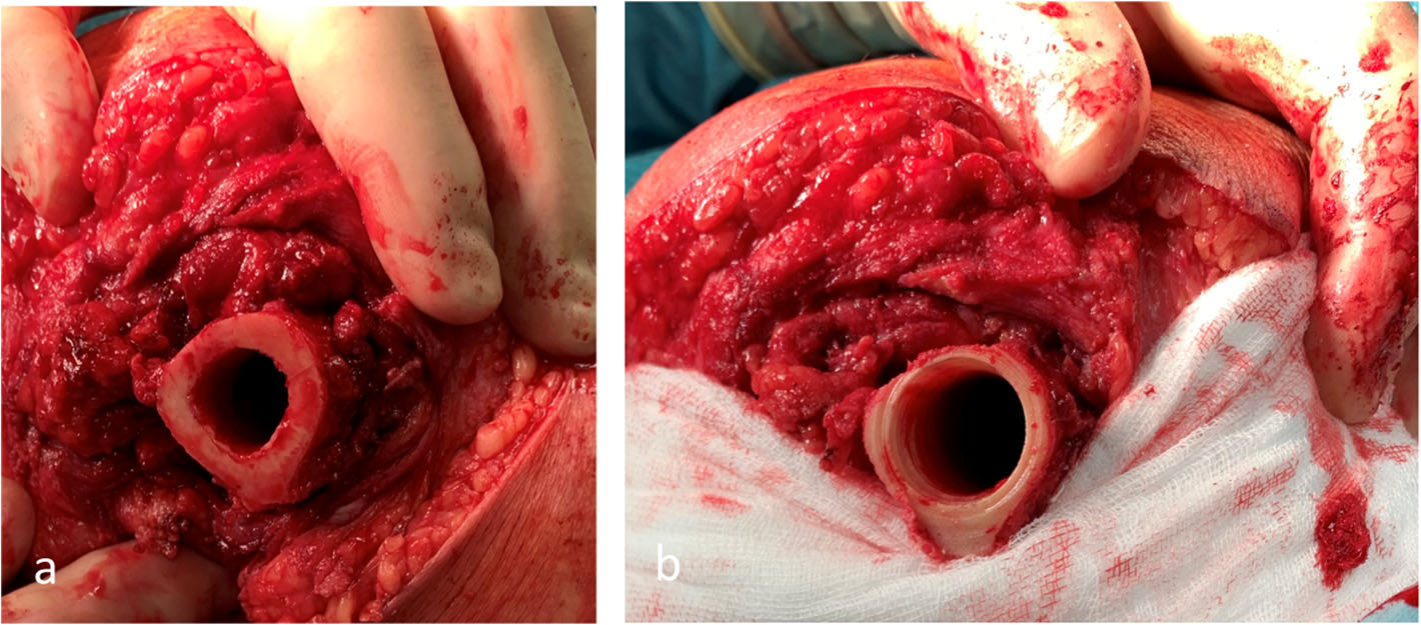
**a** Illustration of the distal femur after flexible reaming. **b** After flexible reaming of the femur canal follows rigid reaming of the distal part of the femur which produces an asymmetrical cortex (ventral side thinner than dorsal)

**Fig. 8:**
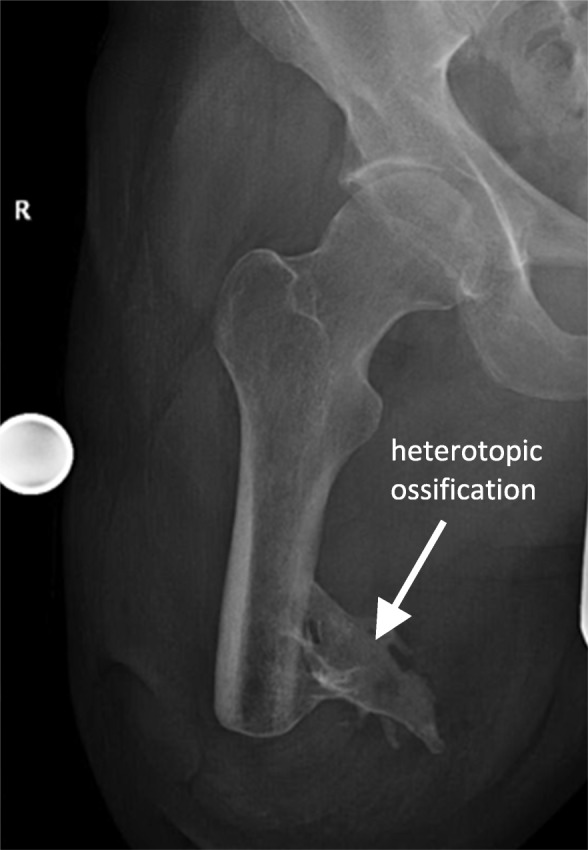
Preoperative x-ray with a 30-mm reference ball to plan an EEP treatment. Large ossification depicted at the distal medial femur

Furthermore, the surgical technique may influence long-term changes in the femoral cortex. It requires rigid, conical drilling at the distal end of the residual bone immediately prior to implantation of the endo-fix stem [4, 10] (Fig. 7). This surgical step is necessary to adapt the femoral medullary canal to the shape of the implant (Fig. 1a). The reduced bone substance presumably leads to a reduction of the bone blood flow, which could further affect undisturbed osseointegration. In the absence of contact osteogenesis (tripod-free area of the implant) and partially thinned osseous structure, a progressive atrophy of the bone in this area can be detected [31, 32]. On the other hand, excessive bone formation at the distal femur (Fig. 5) was also observed in patients who had undergone the same surgical technique. This could be the result of minimal vibrations of the implant at the distal implant-bone interface and, according to Wolff’s law, could result in increased bone formation in this area by directly affecting the bone at the implant-bone interface. A further cause for the distal osseous increase in the bone matrix could be the retention of small bone residues (drill meal), which become progressive in size and visible as heterotopic ossifications. In addition, some heterogeneous oscillations already exist preoperatively (Fig. 8) [33].

The three-dimensional tripod surface serves as an elastic intermediate layer and should enable smooth transition between the different elasticity modules of bone and implant [15, 34, 35]. In this way, the introduced forces at the implant-bone interface are split [13, 15, 36, 37] in order to reduce relative movements between implant and bone, so the formation of a tissue interface and ultimately an implant failure should be prevented [38, 39]. Nevertheless, chronic osteomyelitis can occur. The most severe form of this osteomyelitis, grade IV, describes a diffuse osteomyelitis involving the entire bone, which finally ends in bone necrosis [40]. Osteomyelitis is also classified histologically [41, 42]. A distinction is made between acute and chronic forms. Acute osteomyelitis is characterized by the detection of intramedullary neutrophil granulocytes with a clumped chromatin structure as a sign of apoptosis and with optically empty osteocyte lacunae in the sense of bone necrosis [41, 42]. Chronic bacterial osteomyelitis is characterized histopathologically by spongy bone tissue with reactive bone formation and focal inflammatory bone resorption as well as by highly fibrosed medullary spaces with inflammatory infiltrate [41, 42]. There is no corresponding study on the prosthesis system we use. However, it is conceivable that these aspects could be found in histological examinations of the bone from the distal femur. So, the bacterial colonization of the soft tissue stoma could also be responsible for the resorptive processes in the distal bone region [16].

Besides reducing the relative movements between implant and bone by the tripods to avoid a connective tissue interlayer, the approximation of the elasticity modules also serves to prevent aseptic implant loosening “(https://de.wikipedia.org/wiki/Elastizitätsmodul)”. Go Yamako reported that by modifying the implant stiffness, approximating the elastic modulus of bone, bone resorption could be reduced. At the same time, this leads to increased stress at the bone implant interface, accompanied by reduced strength of the bone implant connection [25]. Another prospective study examining CT data sets of the femur after fitting a total hip replacement supported this finding [43].

Muscular atrophy as well as partially incomplete or missing muscle insertions also influences the processes of bone change. These processes lead to a reduced cortical bone structure due to the missing muscular insertions and ultimately to mineral reduced bone quality and subsequent atrophy [8, 44, 45]. This circumstance was not considered in this follow-up examination.

The laws established by Julius Wolff for the analysis of the interaction of bone and Endo-Fix-Stem are the central component on which the assumptions regarding cortical changes in the context of treatment with Endo-Exo-Prosthetics are based. Thus, atrophy at the distal end of the femur could most likely be explained by the missing load bearing at the distal implant-bone interface (area not covered by tripods) according to the stress shielding and osseous hypertrophy in the same area by an increased load transfer between prosthesis and bone, presumably caused by vibrations or possibly by a randomly high press-fit anchorage in this area, according to Wollf’s law [13].

There are several limitations to this study. First, although all patients were supposed to come to all pre-defined follow-up appointments, some patients missed one or more of the scheduled appointments (Table 2). Second, the use of the flap until 2009 (*n* = 14) made distal measurements problematic. Depending on the intraoperative positioning of the flap, this could cover areas either distal-medial or distal-lateral in the a.p. x-ray.

Third, the rotation of the femur while performing x-rays may have influenced the measurements of the cortical thickness. It is known that even small degree deviations in the rotation of the thigh can lead to different cortical thickness due to the asymmetrical form of the femur. Lastly, the weight bearing status of the patients has not been clearly documented in the charts. There could be a correlation between the weight bearing status and the osseous change processes.

Despite all abovementioned limitations, this is, to our best knowledge, the first study measuring changes of cortical thickness in transfemoral amputees treated with EEP. In addition, our study presents the longest follow-up data for patients treated with this implant.

## Conclusion

Based on these investigations and their critical assessment, both bone formation, bone resorption, and growth processes can be observed. The weight transmission between implant and bone, the surface structure of the implant, the amputation level as well as the muscle pull from the outside onto the bone, and the surgical technique, as well as the bacterial colonization, are relevant for the bony changes. Even though our results did not show any significant difference with regard to cortical changes in the load-bearing, diaphyseal region of the bone, we assume, as already shown by other authors (see above), there will be a consecutive increase in cortical bone mass in areas with increased force transmission between bone and prosthesis, and that bone resorption will occur in areas that are not amenable to direct force transmission at the bone-implant interface.

It has not yet been conclusively clarified in detail which process (force transmission, obligatory bacterial colonization, surgical technique, axle load, etc.) leads to hyper- or atrophy. However, the laws established by Julius Wolff and the stress shielding caused by the change from pressure to pull are the central components in the analysis of the interaction of bone and endo-fix stem. They are based on assumptions regarding cortical changes within the framework of the treatment with endo-exo prosthetics and ensure long-term success with sufficient press-fit anchorage. However, further scientific questions have to be investigated.

A lifetime of more than 15 years, accompanied by a high level of patient satisfaction compared to socket wearers, shows that the care concept using transcutaneously derived, osseointegrated prosthesis systems (TOPS) for transfemoral amputees represents a good treatment alternative.

## Data Availability

The data sets generated and/or analyzed during the current study are available at Hannover Medical School repository at the trauma department.

## Abbreviations

TOPS: Transcutaneous osseointegrated prosthetic systems
EEP: Endo-exo prosthesis
mm: Millimeter
a.p.: Anterior posterior
OPRA: Osseointegrated Prostheses for Rehabilitation of Amputees
OIP: Osseointegration prosthesis
*p*: *P* value
E. g.: Exempli gratia
*n*: Number of patients
